# Trends in Teenage Pregnancy Before, During, and After the COVID-19 Pandemic: A Retrospective Study From a Greek Public Hospital (2015-2024)

**DOI:** 10.7759/cureus.91444

**Published:** 2025-09-01

**Authors:** Pelagia Ziti, Chrisi Christodoulaki, Evangelos Antoninis, Vasileios Antoniadis, Vaia G Sarli, Nikolaos Machairiotis, Theofanakis Charalampos, Konstantinos Louis, Ioannis Tsakiridis, Themistoklis Dagklis, Periklis Panagopoulos, Dimos Sioutis

**Affiliations:** 1 Third Department of Obstetrics and Gynecology, Aristotle University of Thessaloniki, Medical School, Hippokrateio Hospital, Thessaloniki, GRC; 2 Third Department of Obstetrics and Gynecology, National and Kapodistrian University of Athens, Medical School, Attikon University Hospital, Athens, GRC; 3 Department of Internal Medicine, General Hospital of Nea Ionia ‘Konstantopouleio-Patision', Athens, GRC; 4 Department of General Surgery, General Hospital of Athens ‘Evangelismos’, Athens, GRC

**Keywords:** covid-19 pandemic, mode of delivery, public health facilities, social and economic factors, teen pregnancy

## Abstract

Background: Teenage pregnancy, which occurs between the ages of 10 and 19, is a cause for concern in the health and social sectors. The present study investigates trends in teenage pregnancy in a university hospital in Greece over a 10-year period, subsequent to the changes in the prevalence of teenage pregnancy and mode of delivery before, during, and after the COVID-19 pandemic.

Methods: A retrospective observational study was conducted based on the recorded data of deliveries from the Obstetrics Department of Attikon University Hospital for the decade 2015-2024. Trends in teenage pregnancies were analyzed across three time periods: before the pandemic (2015-2019), during the pandemic (2020-2022), and after the pandemic (2023-2024). The mode of delivery was also examined.

Results: Teenage pregnancies accounted for 537 (7.3%) out of 7,340 total births. During and after the pandemic, while total births decreased, the proportion of teenage pregnancies increased, reaching 57 (16.4%) in 2024, although the absolute number remained stable. In 2024, 36 (63.1%) adolescent births were via cesarean section, compared to 30 (37%) before the pandemic.

Discussion: The findings highlight the potential long-term social and economic impact of the COVID-19 pandemic on adolescent reproductive health, as well as changes in delivery mode. There is a need to implement specific reproductive health interventions to ensure access to antenatal care and family planning.

## Introduction

Teenage pregnancy is pregnancy that occurs in girls between the ages of 10 and 19 years. Specifically, 1.5 per 1,000 girls aged 10-14 years in the world are projected to give birth in 2022, with the highest rates occurring in Sub-Saharan Africa, Latin America, and the Caribbean [1]. As for Greece, according to the data provided by the Hellenic Statistical Authority, there is a sharp drop in births from 1980 to 2023, from 148,134 births to 71,455 births, about half (52%). Similarly, there is also a decrease in births to teenage girls aged 15-19 years, from 18,446 to 1,936 over 40 years, with the proportion of teenage pregnancies falling from 12% to 2.7% [2]. Teenage pregnancy is strongly associated with social and economic factors, such as limited access to public health facilities, increased vulnerability due to low income and educational level, and lack of sex education programs in schools, where it increases the likelihood of incomplete or incorrect use of contraceptive methods [3,4]. Similarly, sex education programs that encourage abstinence appear to be associated with high rates of teenage pregnancy, while not successfully reducing teenage births [5]. Teenage pregnancies are considered high-risk pregnancies, as they have more perinatal complications, such as low birth weight, preterm births, and low Apgar scores. Associated risk factors include low basic metabolic index (BMI), smoking, drug, or alcohol use during pregnancy. In Europe, since 2001, several family planning and easier access to sexual and reproductive health services have been successfully implemented [6,7]. However, during the coronavirus 2019 (COVID-19) pandemic, socioeconomic inequalities and access to health services and education worsened, resulting in an increase in teenage pregnancies [8,9]. The present study records 10 years of teenage deliveries and aims to observe the impact of the COVID-19 pandemic on teenage pregnancies by recording the number of teenage pregnancies before, during, and after the pandemic, as well as examining the mode of delivery.

## Materials and methods

This was a retrospective observational study of teenage pregnancy rates and mode of delivery. The study was conducted at Attikon University Hospital in Attica, Greece, which at the time of COVID-19 was designated as a reference hospital for COVID-19. From the maternity registry of the Obstetrics Department of the Attikon University Hospital, the number of teenage pregnancies in the period 2015-2024 was recorded. Specifically, deliveries were divided into three categories: the time period 2015-2019 (before the COVID-19 pandemic), 2020-2022 (during the pandemic), and 2023-2024 (post-pandemic). The 537 deliveries involving adolescent pregnancies of 10-19 years of age out of a total of 7,340 deliveries were studied.

The deliveries of teenage pregnancies counted were those that resulted in live births, including both singleton and multiple births. The results were separated according to the mode of delivery: vaginal delivery, cesarean section (C-section), or vacuum-assisted delivery. Approval for the study was obtained from the Ethics Committee of Attikon University Hospital, Attica, Greece (approval no: 326/05-05-2025).

Descriptive statistics of the results were performed. Percentages for the mode of delivery were calculated, and teenage pregnancies were compared with the total percentage of births per year. All proportions are reported in the format n (%).

## Results

A total of 7,340 live births were recorded at the obstetrics clinic of Attikon University Hospital between 2015 and 2024. Of these, 537 (7.3%) were teenage pregnancies (ages 10-19 years). Within the decade, the absolute number of teenage pregnancies remained relatively stable, ranging from 46 (4.9%) in 2018 to 63 (5.1%) in 2016. Over the years, the proportion of teenage pregnancies increased, reaching its highest in 2024 with 57 (16.4%) of total births. From 2015 to 2019 (pre-pandemic), teenage pregnancies accounted for the following proportions of births: 53 (4.8%) in 2015, 63 (5.1%) in 2016, 49 (4.6%) in 2017, 46 (4.9%) in 2018, and 53 (6.5%) in 2019. During the pandemic period, the rates increased to 47 (9.8%) in 2020 and 57 (10.6%) in 2021. After the pandemic, the highest figures were recorded: 56 (15.6%) in 2023 and 57 (16.4%) in 2024, even though the total number of teenage pregnancies remained consistent between 53 and 57 cases annually (Table 1).

**Table 1:** Total births and teenage pregnancies during the period 2015-2024. TP: teenage pregnancies, ELSTAT: Hellenic Statistical Authority.

Year	Total births	Teenage pregnancies	Cesarean sections in TP	Vaginal births in TP	Vacuum-assisted births in TP	Total live births in Greece	Total TP in Greece (%)
		TP (%)				ELSTAT	ELSTAT
2015	1,099	53 (4.8%)	20	31	2	91,847	2,249 (2.2%)
2016	1,239	63 (5.0%)	25	35	3	92,898	2,493 (2.6%)
2017	1,067	49 (4.5%)	22	25	2	88,553	2,354 (2.6%)
2018	942	46 (4.8%)	22	23	1	86,440	2,331 (2.6%)
2019	822	53 (6.4%)	30	19	4	83,756	2,306 (2.7%)
2020	480	47 (9.7%)	24	23	0	84,764	2,347 (2.7%)
2021	539	57 (10.5%)	29	27	1	85,346	2,058 (2.4%)
2022	445	56 (12.5%)	30	22	4	76,095	2,021 (2.6%)
2023	360	56 (15.5%)	31	23	2	71,445	2,047 (2.8%)
2024	347	57 (16%)	36	16	5	-	-
Total	7,340	537 (7.3%)	269	244	24		

The hospital’s data were compared with national statistics from the Hellenic Statistical Authority (ELSTAT). Both showed a sharp drop in total births in Greece, from 91,847 in 2015 to 71,445 in 2023, a 22.3% decrease. Teenage pregnancies also declined by 9%, from 2,249 (2.5%) in 2015 to 2,047 (2.9%) in 2023. However, the percentage of teenage pregnancies slightly increased nationally, from 2,249 (2.5%) in 2015 to 2,347 (2.8%) in 2020 during the early pandemic.

**Figure 1:**
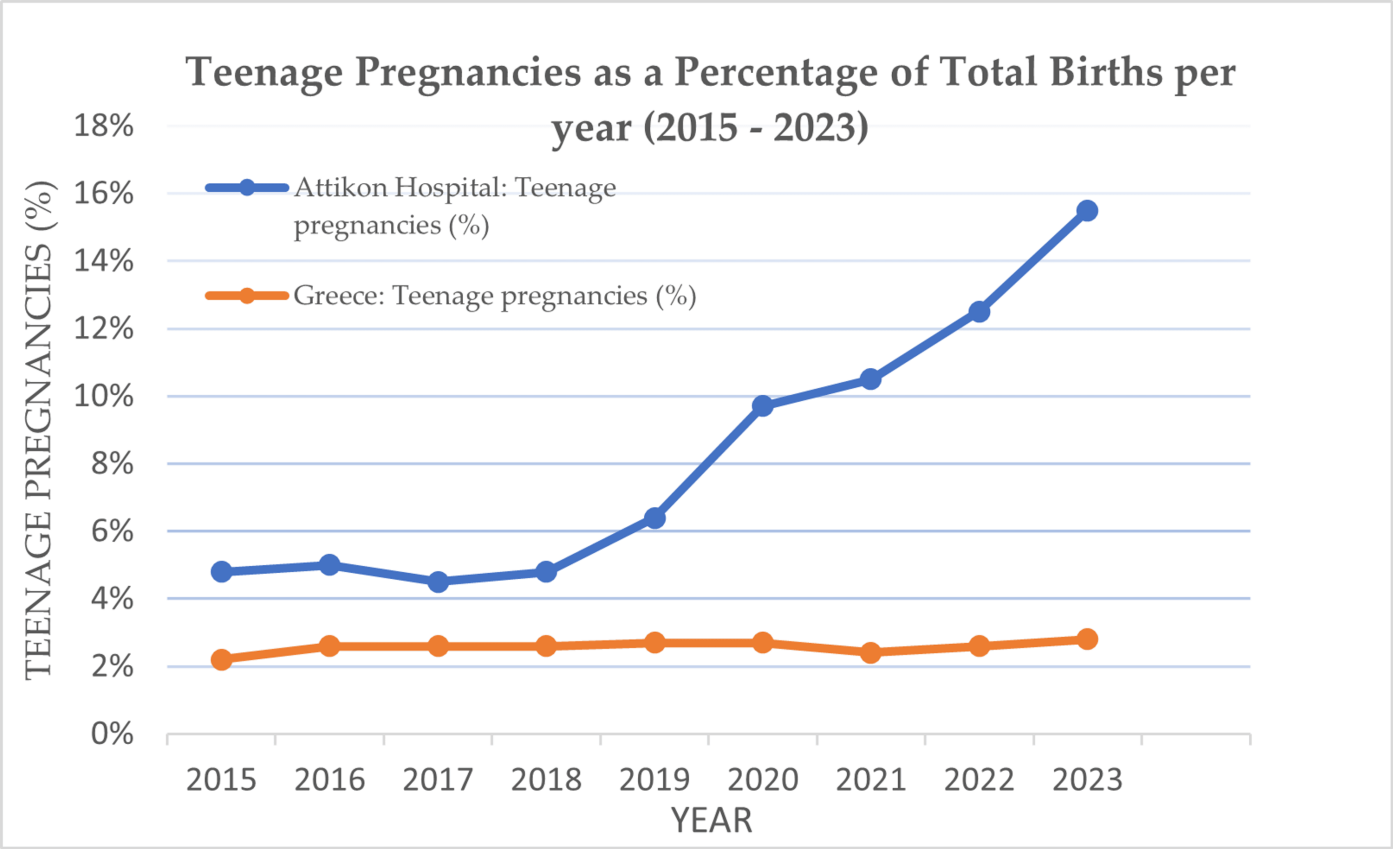
Teenage pregnancies as a percentage of total births (2015-2023).

In contrast, at Attikon Hospital, teenage pregnancies remain stable in absolute numbers over the decade 2015-2023, with 46-63 cases per year. The rate of teenage pregnancies increases gradually, peaking in 2024 at 57 (16.4%). In contrast, total births decline rapidly from 1,099 births in 2015 to 347 in 2024. However, it is not possible to compare the results of the study for 2024, as ELSTAT has not yet published the national statistical data for the latter year (Figure 1).

**Figure 2:**
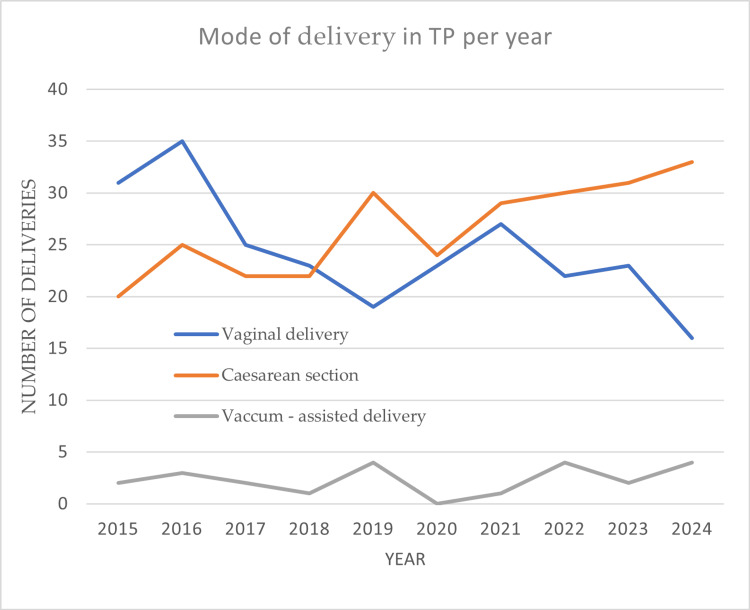
Mode of delivery in teenage pregnancies (TP) per year.

In terms of mode of delivery, there were marked changes in the outcome of teenage births. In total, 269 (50.1%) cases of cesarean section were recorded, compared to 244 (45.4%) cases of normal delivery, and 24 (4.4%) cases of vacuum-assisted delivery. The prevalence of cesarean section use appears to be increasing year by year. In particular, before the COVID-19 pandemic, most pregnancies resulted in a vaginal delivery. The cesarean section rate increased significantly, rising from 119 (37.0%) cases during the pre-pandemic period to 24 (50.9%) in 2020, 29 (53.6%) in 2021, and 30 (53.6%) in 2022 during the pandemic, peaking at 36 (63.1%) in 2024. Vaginal births accounted for 244 (45.4%) of all teenage deliveries, with a sharp decline observed after the pandemic (Figure 2).

Regarding vacuum-assisted delivery, low rates of use were observed, with a total of only 24 cases (4.4%). In conclusion, within the decade, the use of cesarean section prevails over vaginal delivery in adolescent pregnancies.

## Discussion

In the present study, it appears that while total births decrease drastically from 1,099 (2015) to 347 (2024), the number of teenage pregnancies remains relatively stable. The same results are confirmed by the Greek statistical authority, where despite the declining trend in total births in Greece, there is a slight increase observed in 2020 and 2023 in the number of teenage births, at a rate of 2,047 (2.9%) in 2023, indicating a possible change in social or health factors affecting pregnancy at young ages [2].

Before the COVID-19 pandemic, vaginal delivery was the most common method for teenage pregnancies. However, during and after the pandemic, there was a significant rise in cesarean sections, peaking at 36 (63.1%) in 2024. A similar study in Greece found that teenage pregnancies are linked to high rates of cesarean sections and invasive vaginal deliveries [10]. These findings differ from existing literature, which suggests that teenage pregnancies typically have lower cesarean section rates compared to adult pregnancies, possibly indicating a shift in how teenage births are managed [11,12]. One of the reasons in the mode of delivery may be the reduced participation or complete absence of teenage pregnant women in antenatal visits, resulting in an increased risk of adverse neonatal and maternal outcomes [13]. Nevertheless, the high rates of cesarean section in teenage pregnancies may be related to the general increasing trend of cesarean sections in Greece, where approximately 55% of women of childbearing age choose cesarean section over vaginal delivery [14].

The study results reflect global trends showing an increase in teenage pregnancies during the COVID-19 pandemic due to limited access to contraception, school exclusion, and increased exposure to sexual violence in low- and middle-income countries [15,16]. Similarly, high rates of teenage pregnancies continuing after the pandemic (2023-2024) suggest potential long-term social and economic impacts of the crisis, with inequalities in health and education. In the Greek context, due to the strengthening of family planning and access to contraceptive methods (emergency and non-emergency), there is a decrease in the rate of abortions in unwanted teenage pregnancies from 14% to 7.5% [17]. However, teenage pregnancies seem to occur more frequently in populations from lower socioeconomic backgrounds, such as migrant populations and refugees [18], suggesting reduced access to healthcare services.

The findings of our study reflect both localized and global trends in teenage pregnancy patterns across the three defined periods: pre-pandemic (2015-2019), during the pandemic (2020-2022), and post-pandemic (2023-2024). In agreement with the literature, our results highlight how the pandemic functioned as a significant disruptor in access to reproductive health services, education, and social stability for adolescents.

Globally, studies such as those by Okeke et al. and Meherali et al. have demonstrated a marked increase in adolescent pregnancies during COVID-19 lockdowns, primarily due to interrupted access to contraceptive services, closure of schools, and heightened exposure to domestic violence and sexual abuse [9,15]. Our findings support this, as we observed a relative increase in the proportion of teenage pregnancies during and after the pandemic at our center, despite a stable absolute number of cases. This proportional rise is largely attributed to a dramatic decline in total births, mirroring national trends as recorded by ELSTAT.

Importantly, the steady absolute numbers of adolescent pregnancies at Attikon Hospital contrast with the slight national decrease in teenage pregnancies, highlighting the need to consider regional disparities. These may stem from socioeconomic variables such as population demographics, access to health education, and healthcare facility designation.

With regard to the mode of delivery, our findings indicate an increase in cesarean section rates among adolescents, from 37% pre-pandemic to 63.1% in 2024. This rise is consistent with previous Greek studies reporting high cesarean section (CS) rates in adolescent pregnancies [10], but it diverges from broader international trends. For instance, Malabarey et al. and Torvie et al. found that adolescent pregnancies, especially among younger teens, were associated with lower CS rates compared to adults, possibly due to smaller fetal size and fewer comorbidities [11,13].

Our results suggest a deviation from this trend, possibly due to evolving obstetric practices in Greece or the overmedicalization of birth. Kontopanos et al. reported that Greece holds one of the highest CS rates in Europe, often surpassing 50%, and this may influence obstetric decisions irrespective of maternal age [14]. Furthermore, low antenatal visit attendance among adolescents, exacerbated by pandemic-related restrictions, could contribute to emergency or elective CS decisions to mitigate perceived or actual perinatal risks [17].

In contrast, countries with robust family planning services and comprehensive sex education programs reported resilience during the pandemic. Part et al. highlighted how pre-existing access to youth reproductive health services across the EU played a protective role against rising adolescent pregnancy rates during COVID-19 [8]. Greece, however, appears to lack such infrastructure, with a limited number of school-based sex education programs and underutilized family planning services.

Moreover, although our study did not stratify by socioeconomic status or ethnicity, existing literature underscores that adolescent pregnancy in Greece disproportionately affects vulnerable groups, including migrants and refugees [18]. These populations may have faced heightened barriers to healthcare during the pandemic, including language, legal, and financial obstacles, possibly explaining the persistent rates of teenage births at our center.

This study did not examine socioeconomic factors associated with the causes of the trends observed. Limitations of the study are the sample size with reference to a hospital setting, without extending to other regions of Greece, which makes the results less representative of the whole country. In addition, the sample record was collected from the hospital's handwritten birth registers, which may have caused errors or omissions.

## Conclusions

Attikon University Hospital, where this study was conducted, was designated by the Ministry of Health, during the COVID-19 pandemic, as a reference hospital for COVID-19 cases. The present study showed an increasing proportion of teenage pregnancies after the COVID-19 pandemic, despite an overall decrease in births. Furthermore, the increase in cesarean sections in teenage pregnancies suggests changes in obstetric practices or limited access to antenatal care. In conclusion, the findings highlight the need for targeted interventions in sexual education, access to family planning methods. Also, focus should be made on better access to adequate antenatal care for adolescent mothers, giving emphasis to socially vulnerable groups.
